# A-Site mRNA Cleavage Is Not Required for tmRNA-Mediated ssrA-Peptide Tagging

**DOI:** 10.1371/journal.pone.0081319

**Published:** 2013-11-19

**Authors:** Brian D. Janssen, Fernando Garza-Sánchez, Christopher S. Hayes

**Affiliations:** 1 Department of Molecular, Cellular and Developmental Biology, University of California Santa Barbara, Santa Barbara, California, United States of America; 2 Biomolecular Science and Engineering Program, University of California Santa Barbara, Santa Barbara, California, United States of America

## Abstract

In *Escherichia coli*, prolonged translational arrest allows mRNA degradation into the A site of stalled ribosomes. The enzyme that cleaves the A-site codon is not known, but its activity requires RNase II to degrade mRNA downstream of the ribosome. This A-site mRNA cleavage process is thought to function in translation quality control because stalled ribosomes are recycled from A-site truncated transcripts by the tmRNA-SmpB “ribosome rescue” system. During rescue, the tmRNA-encoded ssrA peptide is added to the nascent chain, thereby targeting the tagged protein for degradation after release from the ribosome. Here, we examine the influence of A-site mRNA cleavage upon tmRNA-SmpB activity. Using a model transcript that undergoes stop-codon cleavage in response to inefficient translation termination, we quantify ssrA-peptide tagging of the encoded protein in cells that contain (*rnb^+^*) or lack (Δ*rnb*) RNase II. A-site mRNA cleavage is reduced approximately three-fold in Δ*rnb* backgrounds, but the efficiency of ssrA-tagging is identical to that of *rnb* ^+^ cells. Additionally, pulse-chase analysis demonstrates that paused ribosomes recycle from the test transcripts at similar rates in *rnb* ^+^ and Δ*rnb* cells. Together, these results indicate that A-site truncated transcripts are not required for tmRNA-SmpB-mediated ribosome rescue and suggest that A-site mRNA cleavage process may play a role in other recycling pathways.

## Introduction

Non-stop mRNAs pose a significant molecular quality control problem for all organisms [1]. Non-stop transcripts lack in-frame stop codons and therefore encode incomplete polypeptides that could be deleterious for the cell. Furthermore, ribosomes stall at the 3′-ends of non-stop mRNA because translation termination and subsequent ribosome recycling require an intact A-site stop codon. All bacteria use transfer-messenger RNA (tmRNA) and SmpB to recycle ribosomes from non-stop messages [1], [2]. tmRNA is a bi-functional RNA that acts as both a tRNA and mRNA to “rescue” stalled ribosomes and target the associated polypeptides for rapid degradation [3]. The tRNA-like domain of tmRNA is aminoacylated with alanine and allows recognition of stalled ribosomes [3], [4]. After the nascent peptide is transferred to tmRNA, the non-stop transcript is released from the ribosome and translation resumes using a short reading frame within tmRNA. In this manner, the tmRNA-encoded ssrA peptide is added to the C-terminus of the nascent chain. The ssrA peptide is recognized by several proteases, which rapidly degrade tagged proteins after release from the ribosome [3], [5], [6], [7]. Because the ssrA coding sequence is terminated with a stop codon, the rescued ribosome is able to undergo normal translation termination and recycling. SmpB is a small tmRNA-binding protein that coordinates the tRNA and mRNA functions of tmRNA [8], [9]. The flexible C-terminal tail of SmpB is required for ribosome binding, and recent structural studies indicate that this region mimics the missing A-site codon:anticodon helix on stalled ribosomes [10], [11], [12]. SmpB is also critical for proper presentation of the tmRNA “resume” codon in the A-site after release of the non-stop message [13], [14]. Thus, tmRNA-SmpB acts as a translational quality control system that responds to non-processive protein synthesis. Because the tmRNA-SmpB complex provides stalled ribosomes with a stop codon in *trans*, this process is often termed *trans*-translation in the literature [15].

tmRNA-SmpB also rescues ribosomes that pause at internal sites within full-length messages. SsrA-peptide tagging activity has been reported for translational pauses that occur at clusters of rare codons [16], [17], [18], in response to specific nascent peptide sequences [19], [20], [21], [22], and during acute starvation for amino acids [23], [24]. In some instances, translational arrest results in cleavage of the A-site codon [24], [25], [26]. These results suggest that A-site mRNA cleavage is induced to generate non-stop mRNA and promote tmRNA-SmpB recruitment to paused ribosomes. This model is supported by *in vitro* studies showing that *trans*-translation occurs more rapidly at ribosomes that have no A-site codon [27]. However, A-site cleaved transcripts are only detected in mutants that lack functional tmRNA-SmpB [25], [26]. One explanation for the apparent lack of A-site mRNA cleavage in wild-type cells is that tmRNA-SmpB recycles stalled ribosomes from cleaved transcripts thereby promoting their rapid turnover [25], [26], [28]. Additionally, not all translational arrests induce A-site mRNA cleavage. In several instances, transcripts are degraded to the 3′-leading edge of the paused ribosome, leaving 12 – 18 nucleotides downstream of the A-site codon [21], [23], [26]. Therefore, although truncated mRNA is an important determinant for tmRNA-SmpB activity, it is not clear that transcripts must be truncated in the A-site codon for efficient *trans*-translation.

The 3′-to-5′ exoribonuclease RNase II (encoded by the *rnb* gene) is required for efficient A-site mRNA cleavage in *E. coli* [29]. Translational arrest in *E. coli* Δ*rnb* mutants produces transcripts that are truncated to a position 12 nucleotides downstream of the A-site codon [29]. This +12 truncation site probably corresponds to the “toeprint” of the paused ribosome on mRNA, suggesting that another nuclease(s) degrades transcripts to this position in the absence of RNase II. Notably, RNase II cannot degrade mRNA into the ribosome A site and therefore its role in A-site cleavage must be indirect [29], [30]. We have proposed that RNase II degrades mRNA downstream of the paused ribosome, which then facilitates the activity of the actual A-site nuclease. In accord with this model, A-site cleavage is suppressed by stable mRNA structures that are resistant to degradation by RNase II [29], [31], [32]. In this study, we modulate A-site mRNA cleavage to determine its importance for tmRNA-SmpB mediated ribosome rescue. We find that ssrA-peptide tagging is indistinguishable in *rnb^+^* and Δ*rnb* genetic backgrounds. Moreover, the rates of peptidyl-tRNA turnover from stalled ribosomes are similar in *rnb* ^+^ and Δ*rnb* cells, indicating the ribosome recycling is largely unaffected by the A-site mRNA cleavage process. Together, these results suggest that mRNA degradation to the 3′-edge of the stalled ribosome is sufficient for efficient tmRNA-SmpB rescue activity.

## Materials and Methods

### Bacterial strains and plasmids

All bacterial strains were derivatives of *E. coli* strain X90 and are listed in Table 1. Deletions of *rnb*, *pnp* and *rnr* have been described previously [21]. These alleles were introduced into strains CH12, CH113 and CH2385 by phage P1-mediated generalized transduction [33]. The *rnb rnr* double mutant was constructed by removing the kanamycin-resistance cassette [34] from the Δ*rnb::kan* allele to create CH113 Δ*rnb*, followed by phage P1-mediated transduction of the other gene deletions into the Δ*rnb* background. All other gene deletion constructs were transduced from the Keio collection [35] into strains CH113 or CH113 Δ*rnb*. All strains were subjected to whole-cell PCR to confirm chromosomal structure. Plasmid pHis_6_-YbeL-PP was constructed by amplification of *ybeL-PP* using oligonucleotides **ybeL-his6-Nco** (5′ - TAC CAT GGG CAG CAG CCA TCA TCA TCA TCA TCA TTC TAG TCA TAT GAA CAA GGT TGC TCA) and **pET-Eco** (5′ - CGT CTT CAA GAA TTC TCA TGT TTG ACA GC), followed by digestion with NcoI/EcoRI and ligation to plasmid pET11d.

**Table 1:** Bacterial strains and plasmids.

*Strain or plasmid*	*Description* a	*Reference*
CH12	X90 (DE3)	[40]
CH113	X90 (DE3) *ssrA::cat*, Cm^R^	[40]
CH1002	X90 (DE3) *ssrA::cat* Δrne515::kan, Cm^R^ Kan^R^	[29]
CH1207	X90 (DE3) *ssrA::cat* Δrnb::kan, Cm^R^ Kan^R^	[21]
CH1208	X90 (DE3) *ssrA::cat* Δpnp::kan, Cm^R^ Kan^R^	[21]
CH1214	X90 (DE3) *ssrA::cat* Δrnb, Cm^R^	[21]
CH1916	X90 (DE3) *ssrA::cat* Δrna::kan, Cm^R^ Kan^R^	[21]
CH2385	X90 (DE3) *ssrA*(DD)	[24]
CH2790	X90 (DE3) *Δrnb::kan*, Kan^R^	[21]
CH3138	X90 (DE3) *ssrA(DD) Δrnb::kan*, Kan^R^	This study
CH3139	X90 (DE3) *ssrA(DD) Δrnr::kan*, Kan^R^	This study
CH3153	X90 (DE3) *ssrA(DD) Δpnp::kan*, Kan^R^	This study
CH3295	X90 (DE3) *ssrA::cat Δrnr::kan*, Cm^R^ Kan^R^	[29]
CH3566	X90 (DE3) *ssrA(DD) Δrnb Δrnr::kan*, Kan^R^	This study
CH3574	X90 (DE3) *ssrA::cat rncΔ38::kan*, Cm^R^ Kan^R^	[29]
CH3575	X90 (DE3) *ssrA::cat Δrng::kan*, Cm^R^ Kan^R^	[29]
CH3580	X90 (DE3) *ssrA::cat Δrnb Δrnr::kan*, Cm^R^ Kan^R^	This study
CH4312	X90 (DE3) *ssrA::cat Δrnd::kan*, Cm^R^ Kan^R^	[29]
CH4425	X90 (DE3) *ssrA::cat Δrnd Δrnb::kan*, Cm^R^ Kan^R^	This study
CH4456	X90 (DE3) *ssrA::cat ΔrhlB::kan*, Cm^R^ Kan^R^	This study
CH4463	X90 (DE3) *ssrA::cat Δrnt::kan*, Cm^R^ Kan^R^	[29]
CH4465	X90 (DE3) *ssrA::cat Δrph::kan*, Cm^R^ Kan^R^	[29]
CH4704	X90 (DE3) *ssrA::cat ΔelaC::kan*, Cm^R^ Kan^R^	[29]
CH5363	X90 (DE3) *ssrA::cat Δrnb Δrne515::kan*, Cm^R^ Kan^R^	This study
CH5866	X90 (DE3) *ssrA::cat ΔhrpA::kan*, Cm^R^ Kan^R^	This study
CH5909	X90 (DE3) *ssrA::cat Δrnb rncΔ38::kan*, Cm^R^ Kan^R^	This study
CH5971	X90 (DE3) *ssrA::cat Δrnb ΔelaC::kan*, Cm^R^ Kan^R^	This study
CH6014	X90 (DE3) *ssrA::cat ΔyafO::kan*, Cm^R^ Kan^R^	This study
CH6015	X90 (DE3) *ssrA::cat Δrnb ΔyafO::kan*, Cm^R^ Kan^R^	This study
CH6023	X90 (DE3) *ssrA::cat ΔrnlA::kan*, Cm^R^ Kan^R^	This study
CH6024	X90 (DE3) *ssrA::cat Δrnb ΔrnlA::kan*, Cm^R^ Kan^R^	This study
CH6104	X90 (DE3) *ssrA::cat Δrnb Δrna::kan*, Cm^R^ Kan^R^	This study
CH6105	X90 (DE3) *ssrA::cat Δrnb Δrnt::kan*, Cm^R^ Kan^R^	This study
CH6107	X90 (DE3) *ssrA::cat Δrnb Δrng::kan*, Cm^R^ Kan^R^	This study
CH6766	X90 (DE3) *ssrA::cat Δrnb ΔrhlB::kan*, Cm^R^ Kan^R^	This study
CH7097	X90 (DE3) *ssrA::cat Δrnb Δrph::kan*, Cm^R^ Kan^R^	This study
CH7098	X90 (DE3) *ssrA::cat Δrnb ΔhrpA::kan*, Cm^R^ Kan^R^	This study
CH8873	X90 (DE3) *ssrA::cat ΔrhlE::kan*, Cm^R^ Kan^R^	This study
CH8874	X90 (DE3) *ssrA::cat Δrnb ΔrhlE::kan*, Cm^R^ Kan^R^	This study
CH8875	X90 (DE3) *ssrA::cat ΔdeaD::kan*, Cm^R^ Kan^R^	This study
CH8876	X90 (DE3) *ssrA::cat Δrnb ΔdeaD::kan*, Cm^R^ Kan^R^	This study
pHis_6_-YbeL-PP	T7 expression of YbeL(E159P) containing an N-terminal hexahistidine tag, Amp^R^	This study
pFLAG-(m)YbeL-PP	Expresses FLAG epitope fused to the C-terminal 49 residues of YbeL-PP, Amp^R^	[29]
pKW1	pACYC184 derived vector, Tet^R^	[16]
pKW23	Plasmid pKW1 derivative that expresses tmRNA(DD), Tet^R^	[16]

a Abbreviations used: Amp^R^, ampicillin resistant; Cm^R^, chloramphenicol resistant; Kan^R^, kanamycin resistant, Tet^R^, tetracycline resistant.

### Protein and RNA analysis

His_6_-tagged proteins were purified by Ni^2+^-affinity chromatography as described [36]. Cultures were grown to mid-log phase (OD_600_∼0.5) and harvested over ice. Cells were collected by centrifugation and frozen at −80°C. Frozen cells were broken by freeze-thaw in urea lysis buffer [50% urea − 10 mM Tris-HCl (pH 8.0) − 150 mM NaCl] and lysates clarified by centrifugation at 13,000 rpm for 15 min. Lysates were incubated with Ni^2+^-NTA agarose resin on a rotisserie for 1 hr at room temperature. The resin was washed with 20 mL of lysis buffer supplemented with 20 mM imidazole. His_6_-tagged proteins were eluted with lysis buffer supplemented with 250 mM imidazole. Protein samples were resolved by SDS-PAGE and stained with Coomassie blue or subjected to immunoblot analysis with polyclonal antibodies to the ssrA(DD) peptide. Fluorescent secondary antibodies (anti-rabbit) were obtained from Rockland Immunochemicals. Stained gels and immunoblots were visualized and quantified using the Odyssey infrared imager and software package (LiCor). RNA was isolated and analyzed as described previously [21], [37]. Transcripts were analyzed by northern blot hybridization using [^32^P]-labeled oligonucleotide probe-T7-SD (5′ - GTA TAT CTC CTT CTT AAA GTT AAA C) as described [37]. A-site truncation products were quantified using the Quantity One software package (BioRad). The reported values for percent A-site truncated mRNA represent the mean ± standard error of the mean (SEM) for three to six independent experiments.

### Pulse-chase analysis

*E. coli* cells were grown to exponential phase in MOPS-buffered defined media [38], pulse labeled with 20 µCi/mL of [^35^S]-L-methionine/L-cysteine (MP Biomedicals − 1175 Ci/mmol) and chased with 0.2 mg/mL unlabeled L-methionine/L-cysteine as described [37], [39]. RNA was isolated and run on acid-urea polyacrylamide gels as described [39]. Gels were dried and visualized by phosphorimaging. Radiolabeled peptidyl-tRNAs were quantified using Quantity One, and double-exponential decay equations were fitted to the data to estimate rates of peptidyl-tRNA turnover. Reported values represent average rates ± SEM for two independent experiments.

## Results

### A-site mRNA cleavage is not required for tmRNA-mediated peptide tagging

**Figure 1:**
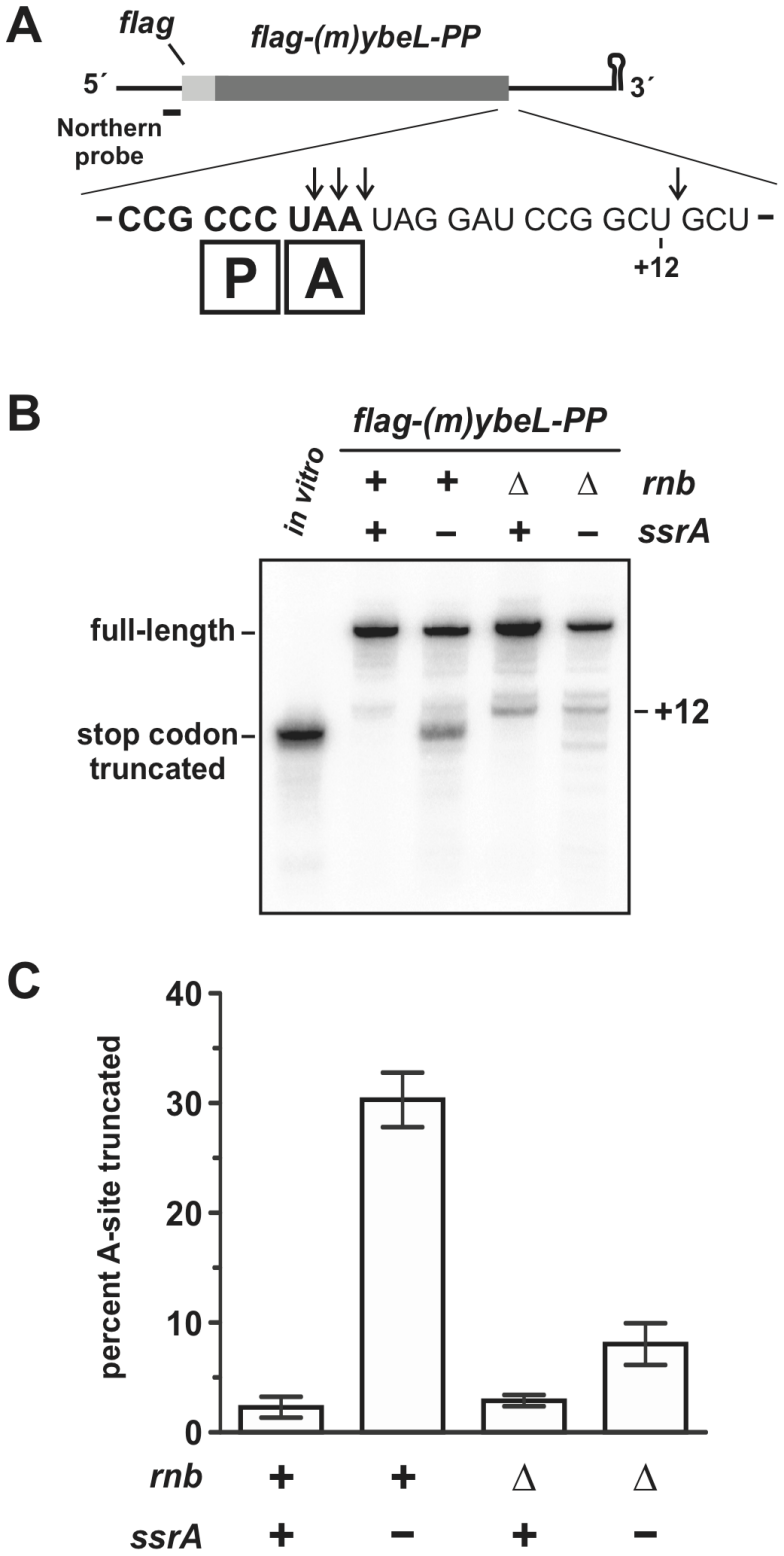
Suppression of A-site mRNA cleavage. **A**) The *flag-(m)ybeL-PP* transcript encodes an N-terminal FLAG epitope fused to the C-terminal 49 residues of *E. coli* YbeL-PP. The position of the P-site and A-site codons during ribosome pausing is indicated by boxed P and A, respectively. Northern blots were hybridized with an oligonucleotide probe that binds both messages just upstream of the initiation codon as indicated. The downward pointing arrows show A-site and +12 cleavage sites as mapped in [29]. **B**) Northern blot analysis of *flag-(m)ybeL-PP* transcripts. Total RNA isolated from cells with the indicated *ssrA* and *rnb* genotypes was probed with a radiolabeled oligonucleotide that hybridizes the 5′-UTR of *flag-(m)ybeL-PP* message. Control transcripts that are truncated at the stop codon were produced by *in vitro* transcription and run as a gel-migration marker in the lane labeled *in vitro*. The migration positions of full-length and truncated transcripts are indicated. **C**) Quantification of A-site truncation products. The percentage of A-site truncated mRNA in each genetic background was determined by quantifying northern blot hybridization signals as described in Methods. Reported values represent the mean ± SEM for at least three independently prepared RNA samples.

The correlation between A-site mRNA cleavage in *ssrA^−^* cells and ssrA-peptide tagging activity in *ssrA* ^+^ cells suggests that these processes are linked functionally. To test this model, we asked whether suppression of A-site mRNA cleavage leads to reduced peptide tagging. We chose the previously characterized YbeL-PP protein from *E. coli* as a model system to study site-specific translational arrest [25], [37], [40]. YbeL-PP carries a C-terminal Pro-Pro nascent peptide motif that interferes with translation termination [40], [41], [42]. As a consequence, the *ybeL-PP* stop codon is cleaved to generate a non-stop message [25], and the nascent chain is tagged with the ssrA peptide [40]. To facilitate the analysis of cleaved *ybeL-PP* transcripts, we used the *flag-(m)ybeL-PP* mini-gene construct, which encodes a FLAG epitope fused to the C-terminal 49 residues of YbeL-PP (Fig. 1A). The Pro-Pro motif induces ribosome arrest in all genetic contexts tested, and A-site cleavage and ssrA-peptide tagging activities are essentially identical for the full-length and mini-gene *ybeL-PP* constructs [25], [29], [37], [40]. Full-length *flag-(m)ybeL-PP* transcripts predominate in *ssrA^+^* cells, whereas approximately 30% of the message is truncated at the stop codon in *ssrA^–^* cells (Figs. 1B & 1C).

RNase II is required for efficient A-site mRNA cleavage activity, and *flag-(m)ybeL-PP* transcripts are truncated +12 nucleotides downstream of the stop codon when expressed in *E. coli ssrA^−^* cells that lack RNase II (encoded by the *rnb* gene) (Figs. 1A & 1B) [29]. Quantification of the stop-codon truncated transcripts indicates that these products are approximately three-fold less abundant in *ssrA^−^* Δ*rnb* cells (8.1±1.9%) compared with *ssrA^−^ rnb^+^* cells (Fig. 1C).

**Figure 2:**
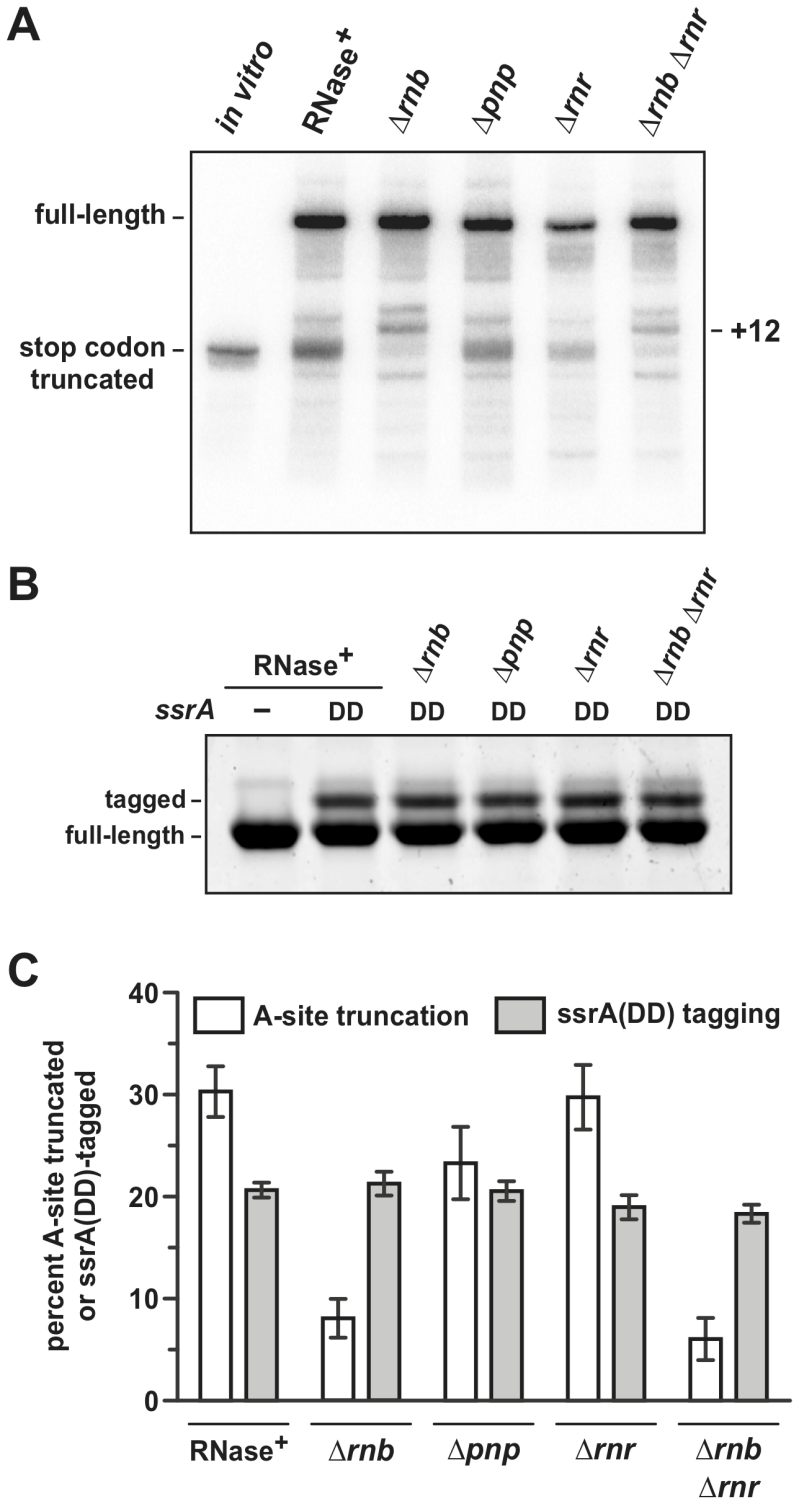
A-site mRNA cleavage is not correlated with ssrA-peptide tagging activity. **A**) Northern blot analysis of *flag-(m)ybeL-PP* transcripts in cells lacking 3′-to-5′ exoribonucleases. Total RNA was isolated from *E. coli ssrA^−^* cells that lack the indicated RNase genes and probed with a radiolabeled oligonucleotide that hybridizes the 5′-UTR of *flag-(m)ybeL-PP* message. The lane labeled *in vitro* contains *flag-(m)ybeL-PP* mRNA that is truncated at the stop codon. The migration positions of full-length and truncated transcripts are indicated. **B**) SsrA(DD)-peptide tagging of His_6_-YbeL-PP. His_6_-YbeL-PP chains were purified from cells of the indicated genetic backgrounds and resolved by SDS-PAGE and stained with Coomassie blue. **C**) Quantification of A-site mRNA cleavage and ssrA(DD) tagging efficiency. The percentage of A-site truncated mRNA was determined by quantifying northern blot hybridization signals as described in Methods. The effect of each RNase gene deletion was examined in an *ssrA^−^* background, and the data (in white bars) represent the mean ± SEM for at least three independently prepared RNA samples. Full-length and ssrA(DD)-tagged His_6_-YbeL-PP chains were isolated from *ssrA(DD)* cells and quantified by densitometry. Tagging efficiency (in gray bars) is reported as the percentage of total chains that carry ssrA(DD) peptides. The presented data represent the mean ± SEM from four independent experiments.

To determine whether diminished A-site cleavage correlates with decreased tmRNA-SmpB activity, we examined ssrA-peptide tagging of full-length YbeL-PP proteins in *E. coli ssrA(DD)* cells. The *ssrA(DD)* allele specifies a tmRNA variant that encodes the ssrA(DD) peptide tag. This modified tag is resistant to proteolysis and ssrA(DD)-tagged proteins accumulate in the cell [3], [5]. Analysis of total His_6_-YbeL-PP protein produced in *ssrA(DD)* cells compared to *ssrA^−^* cells shows that 20±0.7% of the chains are ssrA(DD)-tagged (Figs. 2B & 2C). Somewhat surprisingly, His_6_-YbeL-PP produced in *ssrA(DD)* Δ*rnb* cells is tagged at essentially the same level (21±1.2%) as protein from *ssrA(DD) rnb* ^+^ cells (Figs. 2B & 2C), indicating that the suppression of A-site cleavage activity does not significantly impede tmRNA-SmpB recruitment to stalled ribosomes.

*E. coli* contains two additional 3′-to-5′ exoribonucleases, polynucleotide phosphorylase (PNPase) and RNase R, that play significant roles in mRNA turnover [43], [44]. Therefore, we tested cells deleted for *pnp* and *rnr*, which encode PNPase and RNase R, respectively. Transcript processing is slightly reduced in *ssrA^−^* Δ*pnp* cells (23±3.6%), but unaltered in *ssrA^−^* Δ*rnr* cells (30±3.2%) (Figs. 2A & 2C). Similarly, ssrA(DD)-peptide tagging activity is not significantly altered in Δ*pnp* or Δ*rnr* cells (Figs. 2B & 2C). Cells deleted for both *rnb* and *rnr* exhibit the same mRNA processing as Δ*rnb* single mutants, but tagging activity may be subtly reduced (18±0.9%) compared to *rnb^+^ rnr^+^* cells (Fig. 2A, 2B & 2C). *E. coli* Δ*rnb* Δ*pnp* and Δ*rnr* Δ*pnp* double mutants are not viable [45], [46], therefore we were unable to test other mutation combinations. Taken together, these results show that RNase II plays a role in *flag-(m)ybeL-PP* transcript processing, but has little effect on tmRNA-SmpB-mediated peptide tagging.

### Role of other RNases and RNA helicases in mRNA processing and tmRNA-mediated peptide tagging

**Figure 3:**
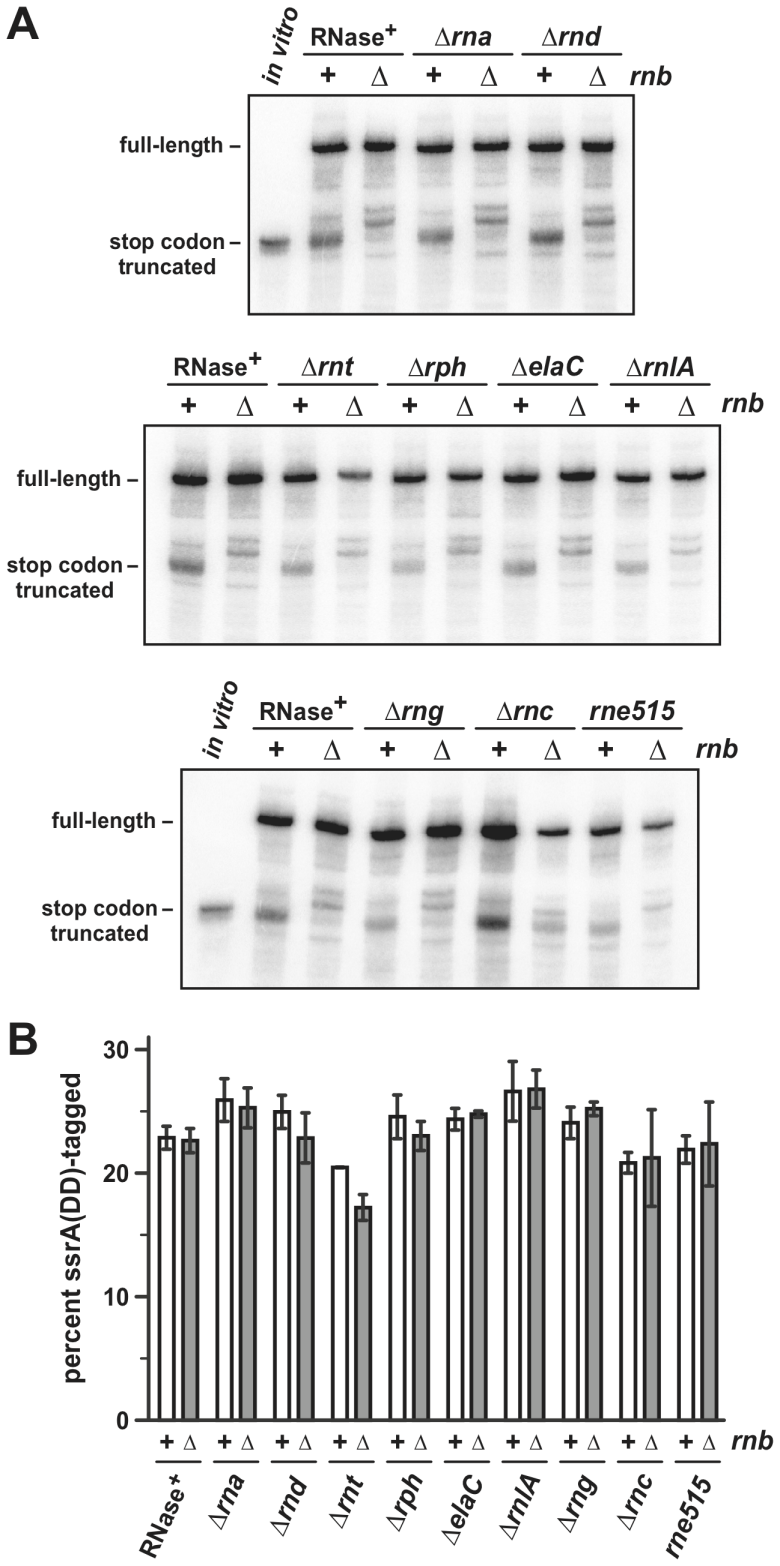
Effect of RNase deletions on mRNA processing and ssrA(DD)-peptide tagging. **A**) Northern blot analysis of *flag-(m)ybeL-PP* transcripts. Total RNA was isolated from *E. coli ssrA^–^* cells with the indicated genotypes and probed with an oligonucleotide that hybridizes to the 5′-UTR of *flag-(m)ybeL-PP* message. The lanes labeled *in vitro* contain *flag-(m)ybeL-PP* mRNA that is truncated at the stop codon. The migration positions of full-length and stop codon truncated transcripts are indicated. **B**) Quantification of ssrA(DD) tagging efficiency. Full-length and ssrA(DD)-tagged His_6_-YbeL-PP chains were quantified by densitometry and tagging efficiency reported as the percentage of total chains that carry ssrA(DD) peptides. Reported values represent the mean ± SEM from two independent experiments.

The presence of +12 truncated transcripts in Δ*rnb* cells suggests that another unidentified RNase degrades mRNA to this position in the absence of RNase II. To identify the enzyme responsible for this activity, we examined mRNA processing in strains that are deleted for known RNase genes. RNase gene deletions were transferred into *E. coli ssrA^−^* Δ*rnb* cells by transduction and the effects on *flag-(m)ybeL-PP* processing were assessed by northern blot analysis. Deletion of genes encoding RNase I (*rna*), RNase D (*rnd*), RNase T (*rnt*), RNase PH (*rph*), RNase Z (*elaC*), RNase LS (*rnlA*) and RNase G (*rng*) has only modest effects on A-site mRNA cleavage in the *ssrA^−^* background and +12 cleavage in the *ssrA^−^* Δ*rnb* background (Fig. 3A). Additionally, deletion of the C-terminus of RNase E (*rne515*), which organizes the multienzyme RNA “degradosome” [47], does not change the pattern of transcript processing (Fig. 3A). However, deletion of *rnc* (encoding RNase III) in the *ssrA^−^* Δ*rnb* background restores A-site mRNA cleavage (Fig. 3A, lowest panel). This latter effect is likely due to ∼10-fold up-regulation of PNPase expression in Δ*rnc* mutants [48]. We have previously shown that PNPase overexpression is sufficient to restore A-site mRNA cleavage in Δ*rnb* mutants [29], suggesting that the Δ*rnc* mutation may have the same effect. Quantification of ssrA(DD)-tagged His_6_-YbeL-PP proteins from the RNase deletion strains showed that tagging activity is not significantly altered in most instances (Fig. 3B). However, ssrA(DD)-tagging efficiency was slightly, but reproducibly, reduced in Δ*rnb* Δ*rnt* mutants (Fig. 3B).

**Figure 4:**
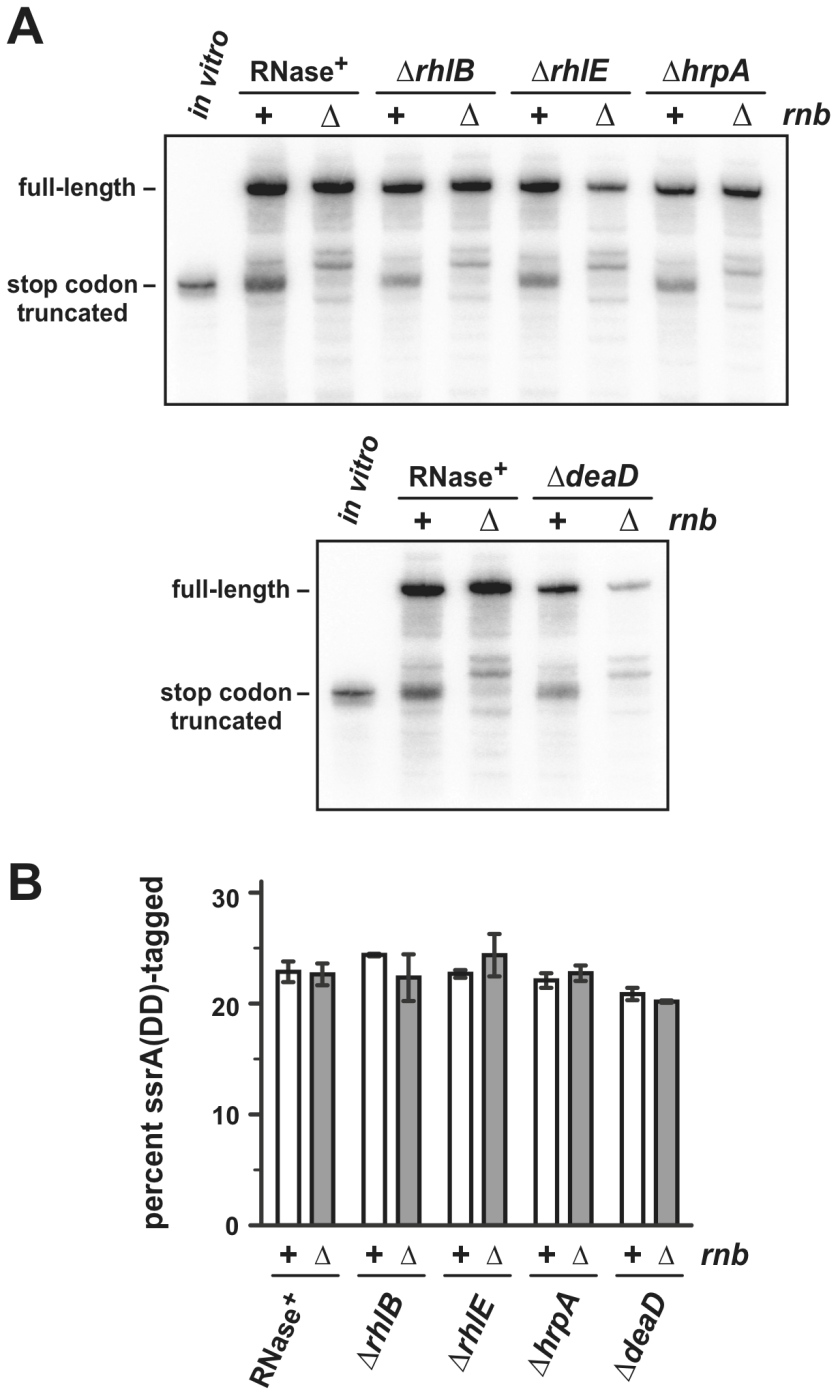
Effect of RNA helicase deletions on mRNA processing and ssrA(DD)-peptide tagging. **A**) Northern blot analysis of *flag-(m)ybeL-PP* transcripts. Total RNA was isolated from *E. coli ssrA^−^* cells with the indicated genotypes and probed with an oligonucleotide that hybridizes to the 5′-UTR of *flag-(m)ybeL-PP* message. The lanes labeled *in vitro* contain *flag-(m)ybeL-PP* mRNA that is truncated at the stop codon. The migration positions of full-length and truncated transcripts are indicated. **B**) Quantification of ssrA(DD) tagging efficiency. Full-length and ssrA(DD)-tagged His_6_-YbeL-PP chains were quantified by densitometry and tagging efficiency reported as the percentage of total chains that carry ssrA(DD) peptides. Reported values represent the mean ± SEM from two independent experiments.

RNA helicases are important for the regulation of mRNA translation in eukaryotic cells and could play similar roles in bacteria [49]. In fact, the DEAD-box helicase, RhlB, is found within the RNA degradosome, where it facilitates mRNA turnover by unwinding secondary structures [50]. Based on these observations, we screened four DEAD-box helicases, RhlB, RhlE, HrpA and DeaD, to determine whether these enzymes influence the A-site cleavage process. We transduced deletion alleles for each helicase into *E. coli ssrA^–^* cells and examined *flag-(m)-ybeL-PP* transcript processing. Each helicase deletion strain showed the same pattern of mRNA cleavage as *ssrA^−^* cells with the full complement of helicases (Fig. 4A). Mutants lacking RNase II (Δ*rnb*) in combination with the individual helicase knockouts did not change the cleavage patterns (Fig. 4A). We also moved the helicase deletions into *ssrA(DD) rnb^+^* and *ssrA(DD)* Δ*rnb* backgrounds, but found that these enzymes have little to no effect on ssrA-peptide tagging efficiency (Fig. 4B). Together, these results indicate that RNase II is required for A-site cleavage and that other known RNases and RNA helicases appear to play no role in this process.

### YafO toxin does not catalyze +12 cleavage during ribosome arrest

**Figure 5:**
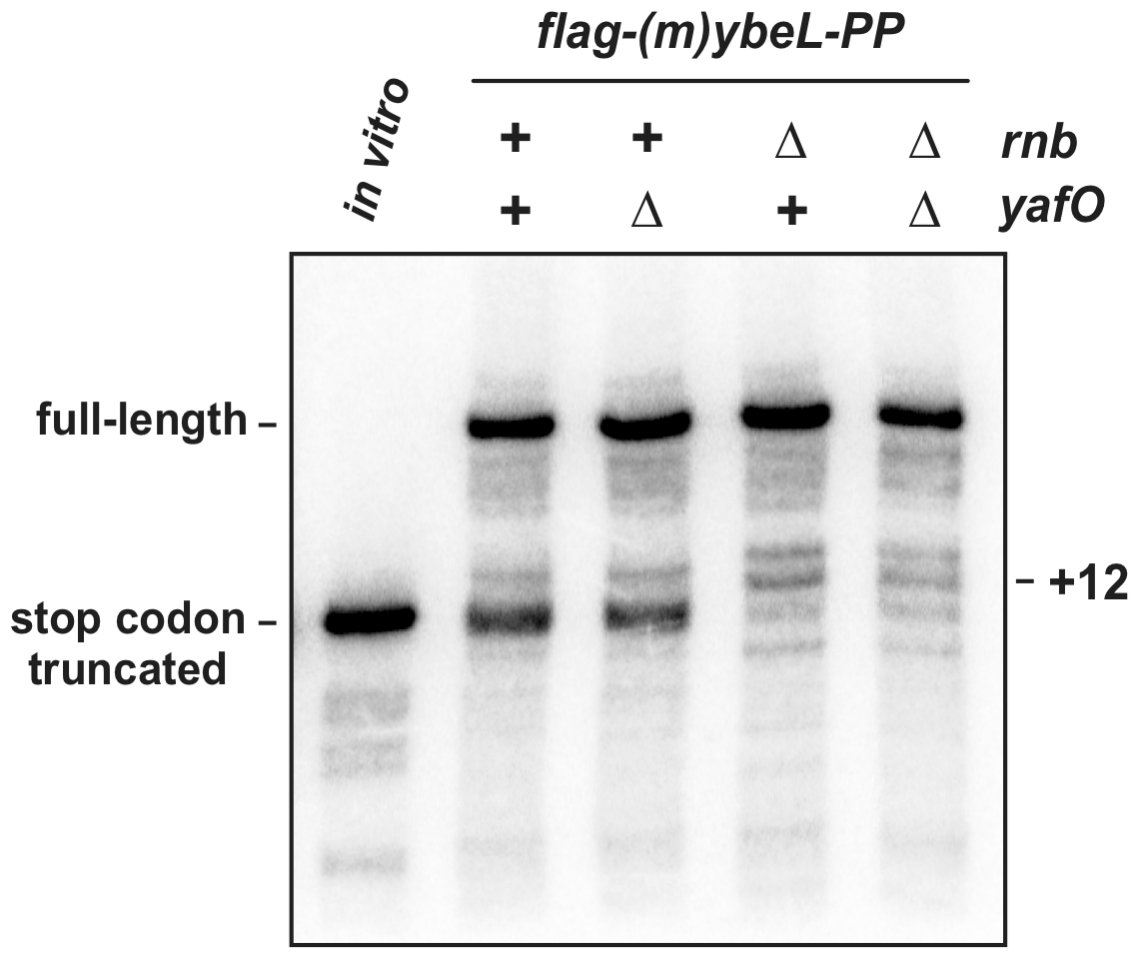
YafO does not mediate +12 processing during translational arrest. Northern blot analysis of *flag-(m)ybeL-PP* transcripts. Total RNA was isolated from *E. coli ssrA^−^* cells with the indicated genotypes and probed with an oligonucleotide that hybridizes to the 5′-UTR of *flag-(m)ybeL-PP* message. The lane labeled *in vitro* contains *flag-(m)ybeL-PP* mRNA that is truncated at the stop codon. The migration positions of full-length and truncated transcripts are indicated.

The results presented above also indicate that none of the tested exoribonucleases are individually required for +12 processing in *ssrA^−^* Δ*rnb* cells. Inouye and colleagues recently characterized a type II toxin/antitoxin (TA) module from *E. coli* that encodes an RNase with an activity that is similar to the +12 cleavage activity described here. YafO acts on translation initiation complexes to cleave mRNA near the +15 position with respect to the P-site AUG initiation codon [51]. This site corresponds to +12 processing in our system, suggesting that YafO may be activated in response to ribosome pausing. We deleted *yafO* in *ssrA^−^* and *ssrA^−^* Δ*rnb* backgrounds and examined the effect on *flag-(m)ybeL-PP* transcript processing, but found no changes in transcript profiles between *yafO^+^* and Δ*yafO* strains (Fig. 5). Thus, the YafO toxin is not required for +12 cleavage during ribosome arrest.

### RNase II activity does not accelerate ribosome recycling

**Figure 6:**
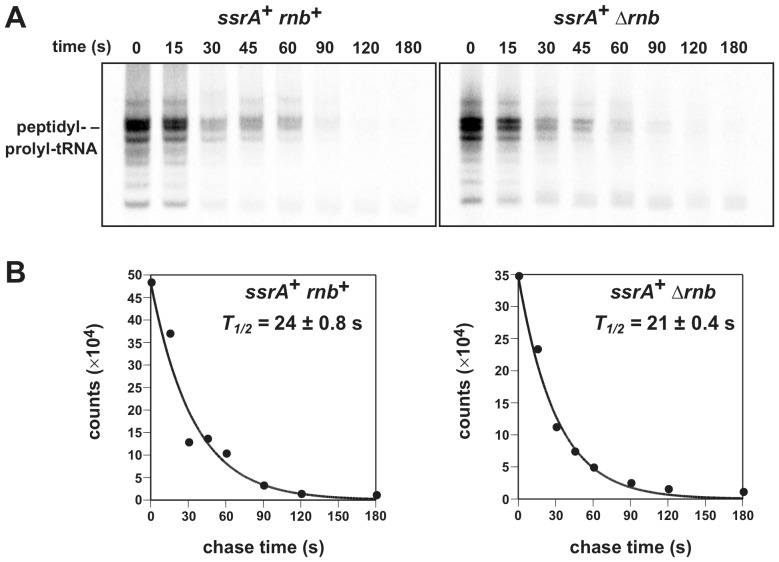
Pulse-chase analysis of ribosome recycling. **A**) Autoradiography of [^35^S]-labeled peptidyl-tRNAs isolated from *rnb^+^* and Δ*rnb* cells. Cells were pulse-labeled and samples taken at the indicated times for denaturing polyacrylamide gel electrophoresis and autoradiography. The band corresponding to peptidyl prolyl-tRNA_2_^Pro^ was identified by northern blot hybridization (not shown). **B)** Double exponential equation fits to experimental data. Because there are at least two distinct pathways for the turnover of peptidyl-tRNA (i.e. release factor mediated termination and tmRNA-SmpB mediated rescue) double exponential decay equations were fitted to the experimental data. Representative fits for *rnb^+^* and Δ*rnb* cells are shown. All experiments were conducted twice and the reported values correspond to the mean ± SEM.

Because A-site cleavage has no discernable effect on ssrA-peptide tagging, this mRNA processing is probably not required for tmRNA-SmpB-mediated ribosome rescue. However, there are at least two other ribosome rescue systems in *E. coli* [52], [53], [54], raising the possibility that A-site cleavage facilitates ribosome recycling through an alternative pathway. Therefore, we measured the rates of paused ribosome recycling in *rnb^+^* and Δ*rnb* backgrounds. Because ribosomes pause during the termination of *ybeL-PP* translation, they carry nascent chains that are covalently linked to P-site tRNA_2_^Pro^ (Fig. 1A). We have previously shown that peptidyl prolyl-tRNA_2_^Pro^ accumulates in response to translational arrest and can be exploited as a biochemical marker of paused ribosomes [37], [39]. We pulse labeled nascent chains with [^35^S]-labeled methionine/cysteine and monitored their turnover during a chase with excess unlabeled amino acids. As reported previously, peptidyl prolyl-tRNA_2_^Pro^ turns-over more rapidly in *ssrA^+^* cells compared to *ssrA^−^* cells (Table 2) [37], consistent with the role of tmRNA-SmpB in ribosome rescue. Somewhat unexpectedly, peptidyl prolyl-tRNA_2_^Pro^ turnover is slower in wild-type *ssrA^+^ rnb^+^* cells compared to the *ssrA^+^* Δ*rnb* cells (Figs. 6A & 6B, Table 2). However, in the *ssrA^−^* background, deletion of *rnb* results in a slightly longer half-life (although almost within error) for peptidyl prolyl-tRNA_2_^Pro^ (Table 2). Together, these data indicate that the A-site mRNA cleavage process has a modest effect on the rate of paused ribosome recycling.

**Table 2:** Peptidyl-tRNA half-lives.^a^

Genetic background	peptidyl prolyl-tRNA half-life (s)
*ssrA^+^ rnb^+^*	24±0.8
*ssrA^+^* Δ*rnb*	21±0.4
*ssrA^−^ rnb^+^*	32±3.0
*ssrA^−^* Δ*rnb*	36±0.9

a *T*_1/2_ values were determined from double-exponential decay equations fitted to data as described in Methods. Reported values are the mean ± standard error of the mean (SEM).

## Discussion

The original model of *trans-*translation postulated that ribosome arrest at the 3′-end of nonstop mRNA is the signal for tmRNA-SmpB recruitment [3]. This conclusion is supported by subsequent *in vitro* studies by Ehrenberg and colleagues. The latter work shows that nascent chain transfer to tmRNA occurs most rapidly when 0–6 nucleotides are present downstream of the P-site codon, and that transfer rates diminish with longer transcripts [27]. Although the initial *in vitro* rate of *trans-*transfer is close to zero when there are 15 nucleotides downstream of the P-site codon (equivalent to +12 processing in our system), these same reactions approach 50% completion after 1 s of incubation [27]. Our results suggest that degradation of mRNA to the 3′-edge of stalled ribosomes is sufficient for tmRNA-SmpB-mediated rescue *in vivo*. Perhaps tmRNA-SmpB induces a slow conformational change in the ribosome that allows *trans-*translation to occur upon prolonged arrest. Structural studies indicate that the C-terminal tail of SmpB interacts with 30S A site, where it is thought to mimic the missing codon:anticodon mini-helix [9], [12], [55]. Therefore, A-site mRNA must presumably be displaced to accommodate tmRNA-SmpB binding. Although there appears to be a discrepancy between the *in vitro* and *in vivo* requirements for *trans*-translation, it is possible that an unknown cellular factor enhances *trans-*translation when transcripts extend beyond the stalled ribosome A site. We note that there are several examples of ribosome arrest that do not induce A-site mRNA cleavage [18], [19], [20], [23], suggesting that longer truncated transcripts represent a major pathway for ribosome rescue. A rigorous test of this model awaits identification of the nuclease(s) responsible for +12 mRNA processing.

Most *E. coli* messages are thought to be first recognized and cleaved by RNase E to produce fragments that are subsequently degraded by 3′-to-5′ exoribonucleases [43]. RNase E preferentially binds monophosphate groups at the 5′-ends of transcripts, and therefore the bulk flow of mRNA degradation proceeds with 5′-to-3′ polarity even though *E. coli* lacks known 5′-to-3′ exoribonucleases. This strategy minimizes the production of translatable non-stop messages during mRNA turnover. Moreover, mRNA turnover is typically processive without the accumulation of decay intermediates. However, the results presented here and elsewhere show that paused ribosomes stabilize partially degraded transcripts that lack 3′-ends [18], [19], [20], [21], [23], [24], [25], [26], [28], [56]. It is unclear whether these fragments represent normal decay intermediates that are stabilized by stalled ribosomes, or whether they accumulate because the 5′-to-3′ degradation pathway is disrupted by queued ribosomes. In either case, paused ribosomes interfere with processive mRNA decay. When we first discovered A-site mRNA cleavage, we proposed that this activity was critical for mRNA turnover because it would accelerate ribosome recycling and expose truncated transcripts to exoribonucleases [25]. Although that original conclusion is not supported by the present study, tmRNA-SmpB activity does indeed hasten mRNA decay. Karzai and colleagues have shown that ribosome rescue leads to rapid degradation of truncated transcripts by RNase R [57], [58]. The results presented here now suggest that +12 cleavage is sufficient for these tmRNA-SmpB-dependent effects on mRNA turnover.

If +12 truncated transcripts are sufficient for the tmRNA-SmpB activity, then what is the functional significance of A-site mRNA cleavage? Because A-site cleavage is only detected in *ssrA* (or *smpB*) mutants, it may be a response to prolonged translational arrest in the absence of ribosome rescue. This model suggests that A-site cleavage could play a role in alternative ribosome rescue mediated by ArfA [53]. ArfA functions as a back-up ribosome rescue system that is only deployed when tmRNA-SmpB is overwhelmed or incapacitated [59], [60]. ArfA binds to stalled ribosomes and induces nascent chain release by recruiting release factor-2 (RF-2) [61], [62]. Because RF-2 activity requires an intact stop codon, it is possible that ArfA binds the 30S A site to mimic a UGA stop codon. Consistent with this model, we have found that ArfA-mediated rescue activity requires an incomplete A-site codon *in vivo* (F.G.S. and C.S.H. unpublished results). However, if A-site mRNA cleavage is required for the ArfA pathway, then *E. coli ssrA^−^* Δ*rnb* cells should be inviable just like *E. coli ssrA^−^* Δ*arfA* mutants [53], [63]. Another possibility is that yet another rescue pathway mediated by YaeJ (ArfB) is upregulated to compensate in these genetic backgrounds [52], [54]. Although YaeJ/ArfB-mediated rescue appears to be quantitatively less important in *E. coli*, it could represent a major rescue pathway in bacteria that lack ArfA. Additionally, because the ribosome arrest studied here is due to consecutive prolyl residues in the nascent chain, it is possible that elongation factor-P could play a role in ribosome-restart to allow RF-mediated termination [64], [65], [66]. Clearly, further study will be required to ascertain the relative importance and functional interactions between these recycling pathways.
